# Elevation of urinary alpha-1-antitrypsin and transferrin excretion in children of patients with nephrolithiasis

**DOI:** 10.2478/abm-2025-0032

**Published:** 2025-10-31

**Authors:** Supawadee Mingmongkol, Nuttiya Kalpongnukul, Poorichaya Somparn, Trairak Pisitkun, Wattanachai Ungjaroenwathana, Piyaratana Tosukhowong, Thasinas Dissayabutra

**Affiliations:** 1 Metabolic Disease in Gastrointestinal and Urinary System Research Unit, Department of Biochemistry, Faculty of Medicine, Chulalongkorn University, Bangkok 10330, Thailand; 2 Systems Biology Center, Faculty of Medicine, Chulalongkorn University, Bangkok 10330, Thailand; 3 Division of Urology, Department of Surgery, Sunpasitthiprasong Hospital, Ubon Ratchathani 34000, Thailand

**Keywords:** antitrypsin, nephrolithiasis, proteomics, transferrin, urinary protein

## Abstract

**Background:**

Children of patients with nephrolithiasis (NL) are highly susceptible to stone development. Abnormal urinary mineral excretion and elevated protein/albumin excretion rates have been reported in disease-free children of patients with NL. Increased protein excretion in these children could be associated with the risk of stone formation.

**Objective:**

Explore the urinary proteomic profiles in children with NL who are highly susceptible to stone development. The suspected urinary proteins were further validated in the urine of children with and without a family history of stone formation.

**Methods:**

Twenty-eight patients with NL (N), 40 volunteers (V), 46 children of patients with NL (NC) and 33 children of volunteer subjects (VC) were enrolled. The 24-hour urine of the participants was collected. Thirty urine samples were randomly selected from each children’s group (NC and VC) to investigate proteins with abnormal excretion. Quantitative proteomic analysis was conducted using tandem mass spectrometry. The levels of bikunin (AMBP), alpha-1-antitrypsin (AAT), transferrin (TF), alpha-2-HS-glycoprotein (fetuin-A), and adiponectin levels were measured in all samples using enzyme-linked immunosorbent assay.

**Results:**

Total urinary protein excretion was increased in both N and NC. Urinary excretion rates for 26 proteins increased and 2 proteins decreased in the NC group compared to the VC group. The urinary excretion rates of AMBP, AAT, and TF in patients with NL and their children were higher than those of control and normal children while fetuin-A was increased only in the NC group. Elevation of urinary AAT and TF was dependent on urinary supersaturation.

**Conclusion:**

Children of patients with calcium oxalate had increased urinary protein excretion, including AAT, TF, AMBP, and fetuin-A, considering the consequences of abnormal urine compositions. Increased excretion of these proteins may impact stone formation in these high-risk childhood members by regulation of renal inflammation, oxidation, crystallization, and crystal growth. We propose that AAT and TF excretion rates are potentially used as indicators for urinary supersaturation in high-risk populations.

Nephrolithiasis (NL) is a common urologic disease that affects middle-aged and elderly populations worldwide. It is well recognized that family members of individuals with NL are at increased risk of developing stones than the general population [1]. A recent study revealed that approximately one-third of NL patients had at least one family member affected by stones, and patients with a positive family history had a higher risk of stone recurrence [2, 3].

Several factors contribute to this abnormality, including dietary, environmental, and hereditary elements. In terms of inheritance, certain genetic diseases such as cystinuria, primary hyperoxaluria, familial hypercalciuria, Dent’s disease, renal tubular acidosis, and deficiency in adenine phosphoribosyltransferase have been found to be responsible for hereditary renal stone diseases [4, 5]. However, most patients with familial renal stones have idiopathic cause.

Increased urinary protein excretion was frequently reported in NL patients [6, 7] and is related to comorbidities such as tubular injury, chronic kidney failure, and cardiovascular disease [8, 9]. Scientists are interested in researching the proteins responsible for lithogenesis. Studies of stone matrix and urine proteomes have been extensively investigated, particularly with proteomic analysis to identify biomarkers of lithogenesis. Tosukhowong et al. reported that Thai patients with NL had urine protein of 460.9 ± 267.4 mg/g of creatinine, and proteomic study showed elevation of several proteins, such as albumin, ferritin, uromodulin, etc. [10]. Boonla et al. reported that 62 proteins including serum albumin, alpha-1-microglobulin/bikunin precursor (AMBP) proteins, and inflammatory proteins, were found in renal stone matrix collected from Thai patients [11]. Other researchers have reported the elevation of inflammatory and fibrotic proteins [12–14]. However, the alteration of these proteins is assumed to occur during the process of inflammation or due to stone formation, rather than being pathogenic proteins.

There is emerging evidence that calcium oxalate stone disease could be hereditary. Sayer Ja. claimed that about 35% of patients with calcium oxalate stones have an affected family member [15]. In Thailand, Sritippayawan et al. reported the relative risk of a family member with kidney stone was 3.2 times higher than in the normal population [16]. Our previous study also found that disease-free children of NL patients had some abnormalities prior to stone formation, particularly urinary electrolytes, glycosaminoglycans, along with elevated urinary supersaturation indices [6, 7, 17]. We hypothesized that these children of stone patients are in a pre-disease or subclinical disease state. These children at risk were expected to have pathogenic urine proteins that play a role in stone disease progression. This study aims to identify proteins with abnormal urinary excretion rates in stone-free children of NL patients through proteomic analysis and to identify the associations between these abnormal protein excretion rates and the risk of stone formation using the supersaturation index. The determination of these abnormal urinary proteins in children with NL patients could be beneficial in understanding the mechanism of familial urinary tract stone disease and developing appropriate urinary biomarkers for stone disease.

## Materials and methods

### Study design

A cross-sectional analytic study was conducted in patients with NL (N) between 18- to 70-year-old who underwent kidney stone removal surgery in the Urologic Division, Department of Surgery, Sunpasitthiprasong Hospital, Ubon Ratchathani Province, Thailand, and their major stone composition was identified as calcium oxalate. Patients with a glomerular filtration rate less than 60 ml/min/1.73 m^2^, urinary tract infection, or those taking any diuretic drugs were excluded from the study. Their children (NC) were enrolled at the same time. Control groups consisted of healthy volunteers (V) living within a 20-kilometer radius from the hospital and children of the volunteers (VC). Healthy volunteers were identified as residents aged 18 to 70-year-old who had a negative history of urinary tract stone, no use of certain drugs that can alter urinary electrolytes and concentration, and normal urinary examination (no red blood cell, white blood cell, crystal, and cast). Children from both groups whose age 6-year-old or older were enrolled with the approval of their parents. Exclusion criteria for participants in participants V, NC, and VC included a positive history of kidney stones or current kidney stones.

All procedures performed in the present study, involving human participation, were approved by the Ethics Committee for Research in Human Subjects in the Fields of Thai Traditional and Alternative Medicine and by the Ethics Committee of the Sunpasitthiprasong Hospital (certificate of approval no. RLC0029/55). Written informed consent was obtained from all participants. Furthermore, parental approval was obtained together with informed consent from the parents of all the childhood participants. Written informed consent was obtained from the parents or legal guardians of all participants.

### The 24-hour urine collection

All participants were asked to collect their whole urine from 8:00 a.m. to 8:00 a.m. the next day (24-h urine). Urine was collected in a disposable sterile plastic container with thymol added as a preservative. The sample was kept on ice during transportation. Plasma was collected, urine volume was measured, and urine sample was tested using a urine strip (Analyticon® Biotechnologies AG, Germany) within 2 h of arrival. All samples were transported by car in a sealed container, with temperatures under 0 degree Celsius to the research laboratory and then stored in the laboratory freezer at –80 °C.

### Urinary protein and creatinine excretion rate

Plasma creatinine, urinary creatinine and protein excretion were measured using electrochemiluminescence (COBAS C6000, Roche, USA) in the Central Lab, King Chulalongkorn Memorial Hospital, and the detection limit was set at 0.01 mg/dL. The results were reported as urinary excretion rate per 24 h (mg/day). Urine samples from participants suspected of incomplete urine collection, undiagnosed stone disease, infection, or hematuria were excluded. The 24 h urine exclusion criteria were: total urine volume less than 0.5 ml/kg/h (12 ml/kg/day), urinary creatinine level less than 27.6 mg/kg/day [18], or positive result for blood or leukocytes detected by urine strip test.

### Proteomic study of urinary protein in pediatric groups

#### Harvest of paired-pooled urine proteins

Urine samples were randomly selected from the NC and VC groups. Thirty urine samples from each group were pooled into 3 sets (10 samples per set). The 3 sets of NC were paired with the 3 sets of the VC for further investigation.

After precipitation of urine samples with 75% ethanol and centrifuged, protein precipitates were obtained, dissolved in lysis buffer, and stored in a refrigerator at 4 °C.

#### Isolation of urinary proteins

The preparation of urinary protein and proteomic study methods was developed by Dr. Trairak Pisitkul [19]. In the present study, a one-dimensional sodium dodecyl sulphate polyacrylamide gel electrophoresis (1D-SDS-PAGE) was used to separate proteins from the harvested urinary protein precipitates. The samples were loaded into 10% SDS-PAGE at 120 mV for approximately 60 min. Coomassie blue was used to identify protein bands. Subsequently, each lane of the paired-pooled protein was cut into 10 identical pieces to reduce the masking effect of high-density proteins.

#### In gel digestion and dimethyl labelling

The gel pieces were washed with 25 mM ammonium bicarbonate (NH_4_HCO_3_) in 50% acetonitrile (ACN). The gel pieces were reduced to 10 mM DL-dithiothreitol (DTT) in 25 mM NH_4_HCO_3_ for 45 min at 56°C and then alkylated with 10 mM iodoacetamide (IAA) for 30 min in the dark at room temperature. After the solution, the gel pieces were washed with 25 mM NH_4_HCO_3_. An equal volume of 100% ACN was added, incubated for 10 min, and then concentrated by speed vacuum centrifugation. For gel digestion, MS-grade trypsin (Promega) was added to the gel pieces at a concentration of 12.5 ng/μl in 25 mM NH_4_HCO_3_, and incubated for 1 h at 4°C. Excess trypsin was removed from the gel pieces. Next, 100 μl of 25 mM NH_4_HCO_3_ was added to the gel pieces and incubated overnight. The eluted peptides were extracted with 30 μl of 50% ACN/0.1% formic acid (FA) in water and then centrifuged for 5 min. The supernatant was transferred to a new tube and concentrated by vacuum centrifugation. The peptides of the NC and VC groups were suspended in 100 μl of 100 mM triethylammonium bicarbonate (TEAB) and labelled with light isotope-coded dimethyl reagent (^12^CH2O and NaBH3CN) and medium isotope-coded dimethyl reagent (^12^CD2O and NaBH_3_CN), respectively. The solutions were incubated at room temperature for 1 h. The labels were quenched by adding 30 μl of 1% (vol/vol) ammonia solution (25%). Peptide solutions from both groups were pooled, mixed and desalted using a C18 stagetip.

#### Liquid chromatography - mass spectrometry (LC-MS/MS) analysis and database search

The peptide samples were dissolved in 0.1% formic acid (Sigma-Aldrich) and subjected to nano-liquid chromatography using the EASY-nLC 1000 (Thermo Fisher Scientific), coupled with a Q Exactive Plus mass spectrometer (Q Exactive Plus Hybrid Quadrupole-Orbitrap, Thermo Fisher Scientific), through an EASY-Spray nanoelectrospray ion source (Thermo Fisher Scientific). The gradient was supplied using an EASY-nLC 1000 UHPLC system and consisted of 5% – 40% acetonitrile in 0.1% formic acid for 50 min, 40%-60% acetonitrile in 0.1% formic acid for 10 min, and 60% – 90% acetonitrile in 0.1% formic acid for 10 min at a flow rate of 300 nl/min. The mass spectrometry (MS) methods included a full MS scan at a resolution of 70,000, followed by 10 data-dependent MS2 scans at a resolution of 17,500. The full MS scan range of 300 to 1600 m/z was selected. Precursor ions with charge states of +1 and greater than +8 were excluded. Fragmentation of precursor ions was performed using higher-energy collisional dissociation (HCD). The MS2 spectra were searched and analyzed using Proteome Discoverer Software 2.0 software (Thermo) based on a database from Uniprot Homo Sapiens with the following search parameters: digestion enzyme was trypsin; the maximum allowance for missed cleavages was 2; the number of maximum modifications was 4; Fixed modifications were carbamidomethylation of cysteine (+57.02146 Da), light and medium dimethylation of N-termini and lysine (+28.031300 and +32.056407 Da); and variable modifications were methionine (+15.99491 Da). The mass tolerances for precursor and fragment ions were set to 10 ppm and 0.02 Da, respectively.

#### Functional annotation analysis

The Database for Annotation, Visualisation, and Integrated Discovery (DAVID) was used for primary analysis of the annotation of reported proteins. In the present study, proteins were classified into 5 pathways, including blood microparticles, plasma membrane, platelet degranulation, acute phase response, and protease inhibition pathways.

#### Validation of candidate protein excretion rates and the associations with urinary supersaturation index

Five significantly elevated proteins in the NC group were selected as candidate proteins for further analysis. Double sandwich technique enzyme-linked immunosorbent assay (ELISA) tests (MyBioSource) were used to quantify protein levels in urine samples from all groups. The results were reported as daily excretion rates (mg/day).

The Tiselius supersaturation index was used to compare the urinary excretion rates of the candidate proteins. The Tiselius index was assessed using the following formula:

$$\text{Tiselius AP}(\text{CaOx})\text{ index }=1.9\times {\text{Ca}}^{0.84}\times \text{Ox}\times {\text{Mg}}^{-0.12}\;{\text{x Cit}}^{0.22}\;{\text{x volume}}^{-1.03}[20]$$

### Statistical analysis

Data were expressed as mean ± standard deviation unless otherwise indicated. All tests were two-tailed and analyzed using a Student’s *t*-test. An ANOVA with Bonferroni post hoc analysis was used for data involving more than two groups. Pearson’s correlation was employed to compare urinary protein excretion and the supersaturation index. Statistical analysis was performed using SPSS v. 22 (IBM, USA). Statistical significance was considered as a *P*-value < 0.05.

## Results

### Baseline characteristics

Regarding the criteria, 82 healthy volunteers were initially recruited, but only 40 subjects were finally enrolled in the research. Most of the candidates were eliminated due to the absence of their children or the informed assent could not be acquired. Five of the initial healthy volunteers were suspected of asymptomatic stone disease due to positive erythrocyte in urine. Alas, there were 146 participants, including 28 patients with NL (N), 40 volunteers (V), 45 children of patients with NL (NC) and 33 children of volunteer subjects (VC). No differences in gender, age, plasma creatinine, urine volume, and urinary creatinine excretion rates were observed between the parent (N vs V) groups and between the children (NC vs. VC) groups (Table 1). Elevated urinary protein excretion rates were observed in the N group compared to the V group (467.2 ± 371.1 vs 68.7 ± 50.0 mg/day in N and V, respectively, *P* = 0.003) and in the NC group compared to the VC group (103.7 ± 46.6 vs 57.7 ± 48.9 mg/day in NC and VC, respectively, *P* = 0.001).

**Table 1.** Baseline characteristics of the participants

	Parental groups			Children groups		
	V	N	*P*	VC	NC	*P*
Gender (% male)	38.1%	71.4%	0.053	51.9%	42.9%	0.504
Age (years-old)	46.8 ± 9.6	44.0 ± 5.3	0.325	17.5 ± 4.7	15.6 ± 5.2	0.773
Plasma creatinine (mg/dl)	0.6 ± 0.2	0.7 ± 0.2	0.290	0.4 ± 0.2	0.4 ± 0.1	0.650
Urine volume (L/day)	1.5 ± 0.4	1.6 ± 0.8	0.531	1.1 ± 0.5	0.8 ± 0.4	0.060
Urine creatinine excretion (g/day)	1.2 ± 0.2	1.3 ± 0.4	0.190	0.8 ± 0.3	0.9 ± 0.4	0.422
Urinary protein excretion (mg/day)	68.7 ± 50.0	467.2 ± 371.1	0.003*	57.7 ± 48.9	103.7 ± 46.6	0.001^#^

1 V, healthy volunteers; N, patients with nephrolithiasis; VC, children of patients with volunteer; NC, children of nephrolithiasis.

* *P* < 0.05 compared to V;

# *P* < 0.05 compared with VC.

### Urinary proteome in children of patients with NL and their volunteer counterparts

**Figure 1.**
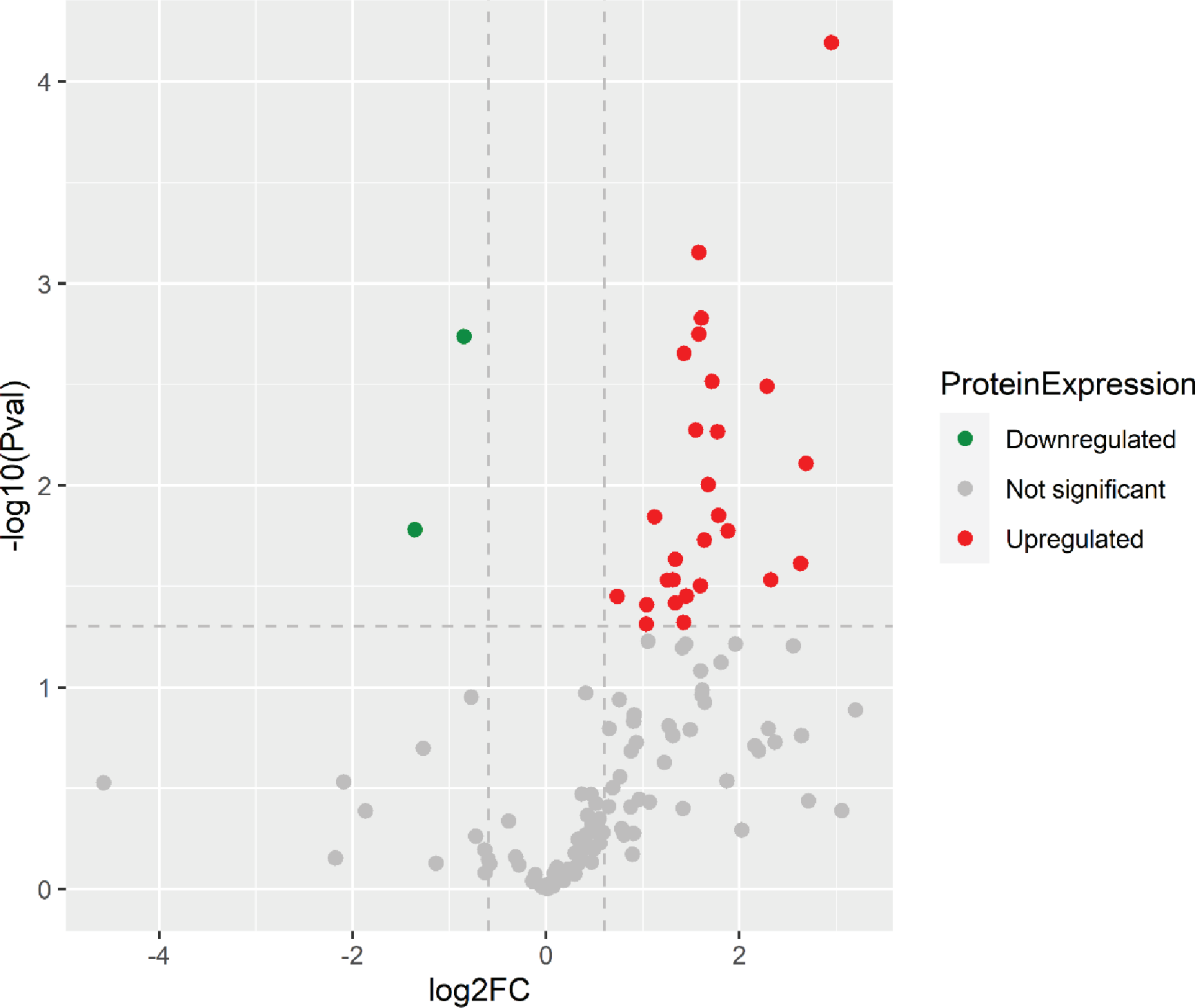
Comparing the urine proteomic profile between children of NL patients and children of volunteers demonstrated an elevation of 26 urinary proteins and the decrease of 2 urinary proteins. NL, nephrolithiasis.

A total of 348 urinary proteins were identified by LC-MS/MS in both NC and VC participants. In the NC group, 28 urinary protein excretion rates were significantly different between the two groups: including 26 proteins up-regulated and 2 proteins down-regulated (Table 2 and Figure 1).

**Table 2.** List of proteins with an abnormal urinary excretion rate in children of patients with NL compared to children of volunteers

Accession no.	List of proteins	Fold change^#^	*P*
	**Proteins with an increased urinary excretion rate**		
4757760	adiponectin	1.60 ± 0.12	0.001
4505529	alpha-1-acid glycoprotein 2	2.28 ± 0.21	0.003
50659080	alpha-1-antichymotrypsin	1.42 ± 0.12	0.002
189163532	alpha-1-antitrypsin	2.32 ± 0.40	0.029
156523970	alpha-2-HS-glycoprotein	2.63 ± 0.43	0.024
530362391	alpha-amylase 1 isoform X1	2.95 ± 0.10	<0.0001
153266841	beta-2-glycoprotein 1	1.78 ± 0.25	0.014
67782358	complement factor B	1.77 ± 0.16	0.005
4503113	cystatin-M	1.58 ± 0.12	0.002
4758092	di-N-acetylchitobiase	1.04 ± 0.20	0.039
148225659	endonuclease domain-containing 1 protein	1.55 ± 0.16	0.005
62122917	filaggrin-2	1.45 ± 0.27	0.035
39995109	ganglioside GM2 activator isoform 1	1.12 ± 0.16	0.014
530395269	hemopexin isoform X1	1.88 ± 0.27	0.017
295986608	immunoglobulin lambda-like polypeptide 5 isoform 1	1.42 ± 0.29	0.048
40549451	lymphatic vessel endothelial hyaluronic acid receptor 1	1.71 ± 0.15	0.003
578814724	maltase-glucoamylase, intestinal isoform X1	0.73 ± 0.14	0.035
4502085	pancreatic alpha-amylase	1.59 ± 0.28	0.031
289547757	prostate stem cell antigen	2.69 ± 0.31	0.008
4502067	protein AMBP	1.25 ± 0.22	0.029
578839125	putative V-set and immunoglobulin domain-containing protein IGHV4OR15-8-like	1.33 ± 0.25	0.038
4557871	serotransferrin	1.31 ± 0.23	0.029
4502027	serum albumin	1.58 ± 0.10	0.001
4885629	trefoil factor 2	1.04 ± 0.21	0.049
88853069	vitronectin	1.63 ± 0.25	0.019
4502337	zinc-alpha-2-glycoprotein	1.33 ± 0.22	0.023
	**Proteins with decreased urinary excretion rate**		
5031839	keratinocyte proline-rich protein	1.36 ± 0.20	0.002
68563515	keratin, type II cytoskeletal 6A	0.85 ± 0.07	0.017

# Data was shown in log 2-based mean ± SEM. NL, nephrolithiasis.

Functional annotation analysis showed that 11 proteins were involved in blood microparticles, 10 in the plasma membrane, 8 in platelet degranulation, 6 in acute phase response and 4 in protease inhibition.

### Urinary excretion rates of candidate proteins and their correlations with urinary supersaturation index

Of the 26 proteins with an elevated urinary excretion rate in the NC group, 5 proteins previously reported to be associated with NL were selected as candidate biomarkers for validation by ELISA. The proteins chosen were AMBP, AAT, TF, fetuin-A, and adiponectin.

Our results demonstrated that three out of five proteins; including AMBP, AAT, and TF, were significantly elevated in the N and NC groups compared to the V and VC groups, approximately 4 times higher (Table 3).

**Table 3.** Urinary excretion rate of candidate proteins in all groups

	Parental groups			Children groups		
	V	N	*P*	VC	NC	*P*
AMBP (μg/day)	10.3 ± 20.11	28.3 ± 41.2	0.048*	10.15 ± 10.56	25.21 +27.77	0.011^#^
AAT (μg/day)	452.6 ± 431.7	1705.2 ± 1393.9	<0.001*	203.7 +206.6	784.7 +450.8	0.025^#^
Transferrin (μg/day)	9.4 ± 7.5	36.5 ± 29.9	<0.001*	4.8 +3.4	16.6 +9.8	0.043^#^
Fetuin-A (μg/day)	2.92 ± 2.58	7.94 ± 13.57	0.193	2.83 +3.27	4.98 +3.52	0.022^#^
Adiponectin (μg/day)	19.9 ± 18.8	87.2 ± 64.7	0.131	29.0 +13.4	58.8 +43.8	0.998

* *P* < 0.05 compared to V;

# *P* < 0.05 compared with VC.

1 AAT, alpha-1-antitrypsin; AMBP, alpha-1-microglobulin/bikunin precursor; N, patients with nephrolithiasis; NC, children of nephrolithiasis; V, healthy volunteers; VC, children of patients with volunteer.

When comparing the Children groups, the increased excretion rate of fetuin-A was higher in the NC group than in the VC group. There was no difference in the urinary excretion rate of fetuin-A between the N and V groups. The differences in adiponectin excretion rates were not significantly different between each group.

**Figure 2.**
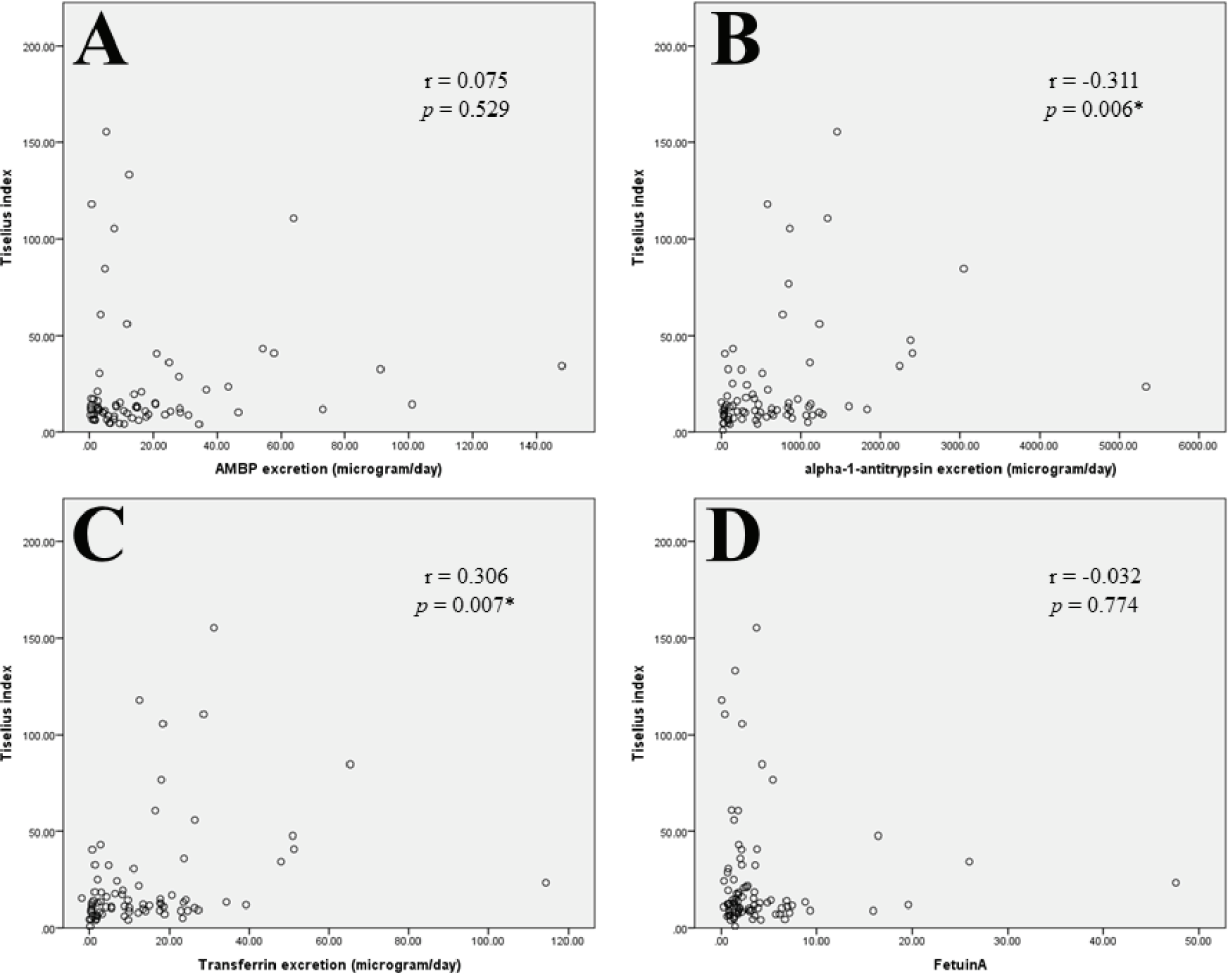
Comparison between the Tiselius urinary supersaturation index (×10-5) and the urinary excretion rate of **(A)** AMBP, **(B)** alpha-1-antitrypsin, **(C)** transferrin, and **(D)** fetuin-A, *P < 0.05. AMBP, alpha-1-microglobulin/bikunin precursor.

Considering protein excretion and urinary supersaturation, urinary excretion rates of AAT and TF were significantly correlated with the Tiselius urinary supersaturation index (Figure 2), while the AMBP, fetuin-A, and adiponectin (data not shown) were not statistically correlated.

## Discussion

NL is a slow progressive disease that can remain asymptomatic for an extended period, with a high recurrence rate, and typically affects close relatives. Having a biomarker for stone development is beneficial in identifying high-risk individuals and for follow-up. Urinary proteins obtained from patients with NL could not be used as biomarkers because most of them were inflammatory proteins or were consequences of inflammation and bleeding, such as proteins involved in immune processes, fibrosis, and fibrinolysis [11]. Therefore, we utilized urine from children of NL patients who were susceptible to identifying pre-existing abnormal protein excretion in this study. Among the 26 elevated urinary protein excretions in the NC group derived from the proteomic study, several proteins were previously reported in patients with NL. Our group also reported an elevation of albumin excretion in patients with NL, and their children was also reported by our group [6]. We selected 5 other proteins that were previously known to be associated with NL for validation in urine derived from all participants. However, only AAT, TF, and AMBP were significantly higher in NC than in VC and in N than V, while fetuin-A was different exclusively in the Children groups.

AAT belongs to the SERPIN superfamily of proteins and contributes to the processes of inflammation, immunomodulation, anti-infection, and coagulation processes [21]. AAT can be used as a biomarker for chronic kidney disease and other inflammatory kidney diseases such as vasculitis [22]. Recent studies have revealed the potential benefits of purified human AAT in hypertension and COVID-19-induced respiratory failure [23, 24]. Elevated urinary AAT was reported in patients with NL [25]. However, AAT is synthesized by the liver and is believed to be a protective protein in response to inflammation and plays a role in the regulation of the serine proteinase activity [26].

Transferrin (TF) is an iron-binding protein produced mainly by liver. Elevation of urinary TF level was presumed to be the result of kidney injury and possibly reflects the downregulation of the tubular epithelial TF receptor [27–29]. Casanova et al. showed that increased urinary TF excretion is a consequence of reduced tubular reabsorption caused by certain drugs, renal hemodynamic change, and tubular damage [30]. Ziqi et al. reviewed that high concentration of calcium oxalate induced ferroptosis of renal tubular cells, leading to reduced TF reabsorption and increased TF excretion [31]. Piyaratana et al. showed that patients with calcium oxalate NL had an elevated urinary transferrin level, which was mitigated after citrate supplementation [10]. According to this, we assume that the elevation of urinary TF is the result of high urinary calcium oxalate or urinary supersaturation, and is renal tubular injury, and less likely to be the cause of stone formation.

Elevations in AAT and TF in NL patients were likely the result of renal tubular inflammation, which is unlikely to be the cause of stone formation. The presence of both proteins could mitigate stone formation per se, by regulation of inflammation, suppression of oxidative stress, and inhibition of ferroptosis. Elevations in urinary excretion in the children of NL patients who have not developed stones could suggest that these children were susceptible to tubular injury and inflammation in relation to high urinary supersaturation.

In the present study, we used urinary supersaturation as a marker of high-risk urinary tract stone formation. It was debatable whether urinary supersaturation is a proper marker for lithogenesis. However, the Tiselius index, which is calculated based on the concentration of calcium, oxalate, citrate, and magnesium, should be able to represent the risk of crystallization and crystal growth of calcium oxalate [32]. Previous studies claimed that excessive urinary supersaturation increases the likelihood of calcium stone formation, but it may be casual factor rather than a causal factor [33, 34]. Recently, Stanislav et al. reported that increased urinary calcium oxalate supersaturation strongly correlated with a higher stone growth rate per year [35]. Regarding this, we assumed that participants with high urinary supersaturation were associated with a high risk of calcium oxalate stone development. Our previous studies demonstrated that children with NL patients had abnormal urinary profiles, including supersaturated urine, remarkably increased urinary excretion rates of calcium, phosphate, oxalate and albumin, comorbid with the decreased urinary excretion rates of citrate and total sulfated glycosaminoglycans [6, 7, 17].

AMBP is a plasma proteinase inhibitor belonging to the lipocalin superfamily. AMBP functions in coagulation, cell growth, and cellular calcium uptake. AMBP plays a role in inflammation and endemic nephropathy and can be used as a biomarker for early tubular dysfunction [36–38], In urine, AMBP acts as a trypsin inhibitor and radical scavenger [39–41]. AMBP attenuates acute kidney injury and inflammation, promotes tissue repair, and contains a crystal growth inhibitory effect [42–44]. The *in vitro* study demonstrated that AMBP strongly inhibits calcium oxalate crystallization [45]. AMBP has been reported to be elevated in patients with NL [46]. In the present study, the urinary AMBP of NL patients and their children was about 4 times higher than that of the normal population but was not correlated with urinary supersaturation.

Fetuin-A, a serum protein mainly synthesized by the liver, mediates serum biomolecules. Increased serum fetuin-A is associated with insulin resistance, diabetes, and bone mineralization [47, 48]. The urinary function of fetuin-A is still controversial, but the C776G polymorphism of fetuin-A may be associated with calcium stone formation [49]. Increased urinary fetuin-A excretion associated with acute and chronic kidney inflammation, interstitial fibrosis, and tubular atrophy [50], suggesting that it was the result of tubular injury, similar to AAT and TF. Furthermore, fetuin-A can bind free calcium and phosphate in urine, preventing calcium crystallization and is considered a urinary tract stone inhibitor [51]. Previous studies revealed lower urinary fetuin-A levels in NL patients with NL [52, 53].

In the present study, urinary fetuin-A in the children of patients with NL was approximately two times higher than in the normal population, but the difference was not significant between the parent groups. A possibility to explain this phenomenon was that fetuin-A production and urinary excretion were decreased by aging [54]. Our study showed that urinary fetuin-A levels, as well as AAT, TF, AMBP, and adiponectin levels in the adult groups were lower than the respective children group. However, urinary AMBP and fetuin-A excretion were independent of urinary supersaturation.

**Figure 3.**
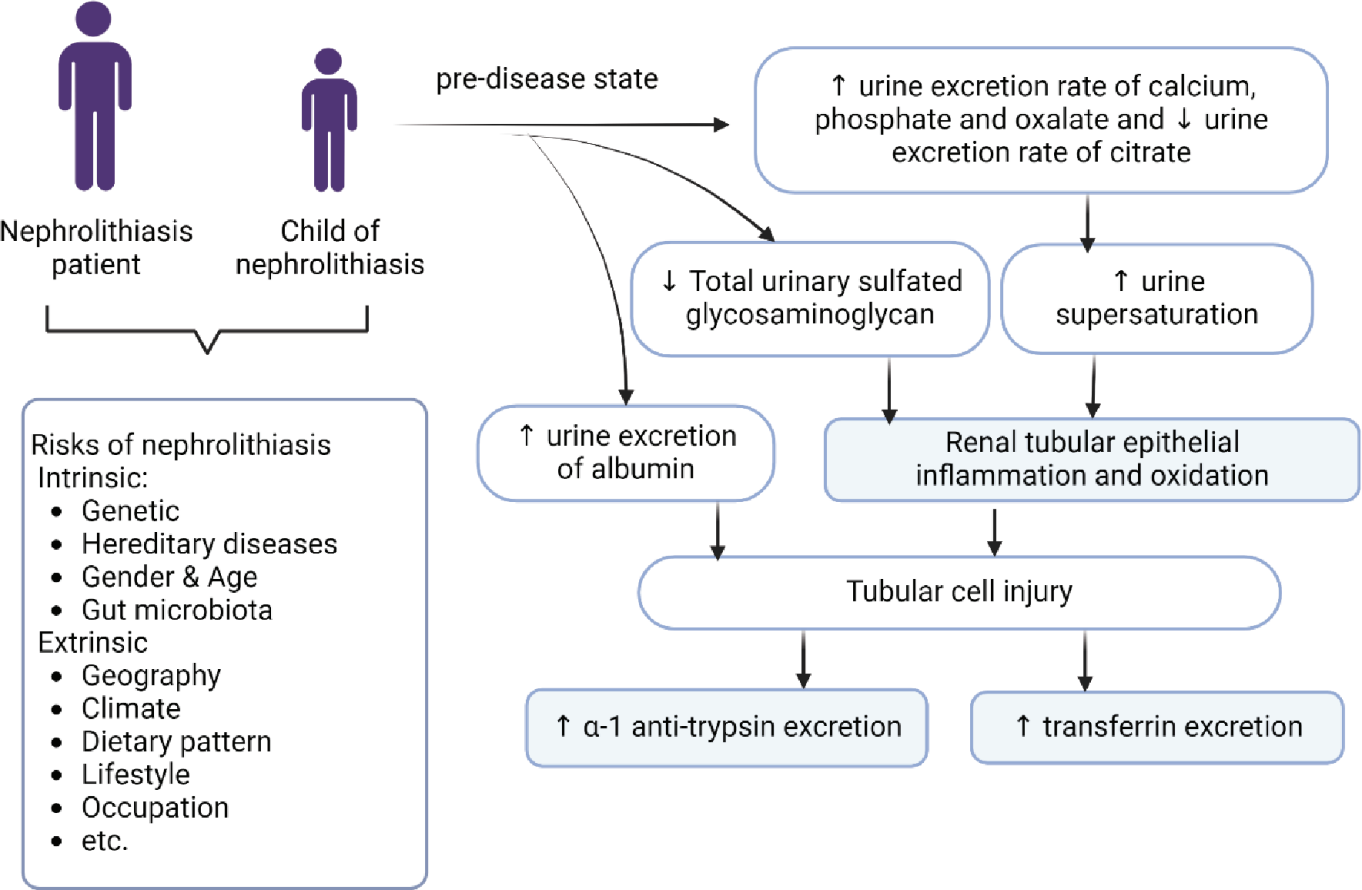
Hypothesis of the elevation of urinary AAT and transferrin protein in children of patients with NL (Created by BioRender.com). AAT, alpha-1 Antitrypsin; NL, nephrolithiasis.

In summary, the present study demonstrated an increase in urinary excretion of total protein, AMBP, AAT, TF, fetuin-A and urinary supersaturation in children with NL patients compared to normal children. These urinary proteins are likely to be the consequences of the prolithogenic state of urine from children in families with stone patients. We hypothesized that children of NL patients who have abnormal urinary mineral excretion, particularly hyperoxaluria, develop subclinical inflammation, oxidation, and tubular injury [55], altering reabsorption, and promoting this protein excretion (Figure 3). Evidently, these proteins could function as anti-lithogenic molecules in suppression of urinary tract inflammation, oxidative stress, crystallization and crystal growth in childhood. Furthermore, children have high levels of other urinary antilithogenic substrates, such as citrate and glycosaminoglycans [56, 57], and less pro-lithogenic factors, such as hypercalciuria, insulin resistance, etc. [58] compared to middle-age and elderly. We assumed that these conditions (high anti-lithogenic molecules and low pro-lithogenic factors in urine) contribute to the prevention of urinary tract stone in the younger population from families with NL.

Nevertheless, the increased urinary excretion of AMBP, AAT, TF and fetuin-A excretion was assumed to be the consequence of urinary abnormalities in children of NL patients. In this study, we were unable to identify the culprit proteins involved in NL pathogenesis. Instead, we propose that these proteins can be used as urine biomarkers for high-risk populations, particularly AAT and TF which are strongly correlate with urinary supersaturation. Another study aims to understand the causal relationship between the reduction of these protein excretion rates and the incidence of calcium oxalate crystals and stones.

## Conclusion

Patients with NL and their children had elevated urinary total protein excretion. A proteomic study in children of NL patients showed increased urinary excretion of 26 proteins. Elevation of AAT and TF excretion associated with increased urinary supersaturation, while AMBP and fetuin-A were independent. This condition was assumed to be a consequence of urine abnormalities and might be beneficial in the prevention crystallization and crystal growth of calcium oxalate in high-risk children. The excretion rates of these proteins, particularly AAT and TF may be used as indicators to evaluate the urinary supersaturation in members of the NL family.
